# Supplementary material for: Subfunctionalization of NRC3 altered the genetic structure of the Nicotiana NRC network
Source: PLoS Genet. 2024 Sep 12;20(9):e1011402. doi: 10.1371/journal.pgen.1011402 (PMC11421798; doi:10.1371/journal.pgen.1011402)
Supplement: S1 Table — (PDF) [file pgen.1011402.s023.pdf]

S1 Table. List of constructs used in cell death assays

Vector backbone	Promoter	protein name	Tag	OD_{600}	Reference
pICH86988	35S	(empty vector)	none	0.5	This study
pICH86988	35S	NRC3 variants	C terminal Myc	0.5	This study
pK7WGF2	35S	Rpi-blb2	N terminal GFP	0.2	[1]
pGWB12	35S	AVRblb2	N terminal Flag	0.1	[1,2]
pTFS40	35S	Pto	C terminal GFP	0.5	[3,4]
pT50	35S	AvrPto	C terminal Flag	0.2	[3,4]
pBIN	35S	R1	none	0.2	[5,6]
pK7WGF2	35S	Avr1	N terminal GFP	0.1	[5,7]
pBIN	GPA2	Gpa2	none	0.5	[8]
pBIN	35S	Rbp1	none	0.5	[8]
pG3101	35S	Rx	C terminal HA	0.05	[9,10]
pG3101	35S	CP	C terminal CBP-SBP	0.05	[10]
pICH86977	35S	Sw5b	none	0.2	[5,11]
pICH86977	35S	Nsm	none	0.1	[5,12]

References

1. Bozkurt TO, Schornack S, Win J, Shindo T, Ilyas M, Oliva R, et al. *Phytophthora infestans* effector AVRblb2 prevents secretion of a plant immune protease at the haustorial interface. Proc Natl Acad Sci. 2011;108: 20832–20837. doi:10.1073/pnas.1112708109

2. Oh S-K, Young C, Lee M, Oliva R, Bozkurt TO, Cano LM, et al. In Planta Expression Screens of *Phytophthora infestans* RXLR Effectors Reveal Diverse Phenotypes, Including Activation of the *Solanum bulbocastanum* Disease Resistance Protein Rpi-blb2. Plant Cell. 2009;21: 2928–2947. doi:10.1105/tpc.109.068247

3. de Vries JS, Andriotis VME, Wu A-J, Rathjen JP. Tomato Pto encodes a functional N-myristoylation motif that is required for signal transduction in Nicotiana benthamiana. Plant J. 2006;45: 31–45. doi:https://doi.org/10.1111/j.1365-313X.2005.02590.x

4. Rathjen JP. Constitutively active Pto induces a Prf-dependent hypersensitive response in the absence of avrPto. EMBO J. 1999;18: 3232–3240. doi:10.1093/emboj/18.12.3232

5. Wu C-H, Abd-El-Haliem A, Bozkurt TO, Belhaj K, Terauchi R, Vossen JH, et al. NLR network mediates immunity to diverse plant pathogens. Proc Natl Acad Sci. 2017;114: 8113–8118. doi:10.1073/pnas.1702041114

6. Ballvora A, Ercolano MR, Weiß J, Meksem K, Bormann CA, Oberhagemann P, et al. The *R1* gene for potato resistance to late blight (*Phytophthora infestans*) belongs to the leucine zipper/NBS/LRR class of plant resistance genes. Plant J. 2002;30: 361–371. doi:10.1046/j.1365-313X.2001.01292.x

7. Du Y, Berg J, Govers F, Bouwmeester K. Immune activation mediated by the late blight resistance protein R1 requires nuclear localization of R1 and the effector AVR 1. New Phytol. 2015;207: 735–747. doi:10.1111/nph.13355

8. Sacco MA, Koropacka K, Grenier E, Jaubert MJ, Blanchard A, Goverse A, et al. The Cyst Nematode SPRYSEC Protein RBP-1 Elicits Gpa2- and RanGAP2-Dependent Plant Cell Death. PLOS Pathog. 2009;5: 1–14. doi:10.1371/journal.ppat.1000564

9. Lu R. High throughput virus-induced gene silencing implicates heat shock protein 90 in plant disease resistance. EMBO J. 2003;22: 5690–5699. doi:10.1093/emboj/cdg546

10. Tameling WIL, Baulcombe DC. Physical Association of the NB-LRR Resistance Protein Rx with a Ran GTPase–Activating Protein Is Required for Extreme Resistance to *Potato virus X*. Plant Cell. 2007;19: 1682–1694. doi:10.1105/tpc.107.050880

11. Spassova MI, Prins TW, Folkertsma RT, Klein-Lankhorst RM, Hille J, Goldbach RW, et al. The tomato gene Sw5 is a member of the coiled coil, nucleotide binding, leucine-rich repeat class of plant resistance genes and confers resistance to TSWV in tobacco. Mol Breed. 2001;7: 151–161. doi:10.1023/A:1011363119763

12. Hallwass M, De Oliveira AS, De Campos Dianese E, Lohuis D, Boiteux LS, Inoue-Nagata AK, et al. The *Tomato spotted wilt virus* cell-to-cell movement protein (NS_M) triggers a hypersensitive response in *Sw-5* -containing resistant tomato lines and in *Nicotiana benthamiana* transformed with the functional *Sw-5b* resistance gene copy. Mol Plant Pathol. 2014;15: 871–880. doi:10.1111/mpp.12144
