# Supplementary material for: Subfunctionalization of NRC3 altered the genetic structure of the Nicotiana NRC network
Source: PLoS Genet. 2024 Sep 12;20(9):e1011402. doi: 10.1371/journal.pgen.1011402 (PMC11421798; doi:10.1371/journal.pgen.1011402)
Supplement: S2 Table — (PDF) [file pgen.1011402.s024.pdf]

S2 Table. List of constructs used in disease resistance assays

Vector backbone	Promoter	protein name	Tag	OD_{600}	Reference
pICH86988	35S	(empty vector)	none	0.5	[1]
pK7WGR2	35S	Rpi-blb2	N terminal RFP	0.2	[2]
pICH86988	35S	NbNRC4	C terminal myc	0.05	[3]
pICH86988	35S	SlNRC3	C terminal myc	0.05	This study
pICH86988	35S	NbNRC3	C terminal myc	0.05	This study
pICH86988	35S	$NN_{PKK}N_{THKI}$	C terminal myc	0.05	This study

References

1. Weber E, Engler C, Gruetzner R, Werner S, Marillonnet S. A modular cloning system for standardized assembly of multigene constructs. PloS One. 2011;6: e16765. doi:10.1371/journal.pone.0016765

2. Bozkurt TO, Schornack S, Win J, Shindo T, Ilyas M, Oliva R, et al. *Phytophthora infestans* effector AVRblb2 prevents secretion of a plant immune protease at the haustorial interface. Proc Natl Acad Sci. 2011;108: 20832–20837. doi:10.1073/pnas.1112708109

3. Wu C-H, Abd-El-Haliem A, Bozkurt TO, Belhaj K, Terauchi R, Vossen JH, et al. NLR network mediates immunity to diverse plant pathogens. Proc Natl Acad Sci. 2017;114: 8113–8118. doi:10.1073/pnas.1702041114
