# Supplementary material for: Subfunctionalization of NRC3 altered the genetic structure of the Nicotiana NRC network
Source: PLoS Genet. 2024 Sep 12;20(9):e1011402. doi: 10.1371/journal.pgen.1011402 (PMC11421798; doi:10.1371/journal.pgen.1011402)
Supplement: S3 Table — (PDF) [file pgen.1011402.s025.pdf]

S3 Table. List of constructs used in co-IP assays

Vector backbone	Promoter	protein name	Tag	OD_{600}	Reference
pGWB12	35S	AVRblb2	N terminal flag	0.1	[1,2]
pGWB555	35S	RFP	none	0.2	[1]
pK7WGR2	35S	Rpi-blb2	N terminal RFP	0.2	[1]
pICH86988	35S	NbNRC3	C terminal myc	0.5	This study
pICH86988	35S	$NN_{PKK}N_{THK}$	C terminal myc	0.5	This study

References

1. Bozkurt TO, Schornack S, Win J, Shindo T, Ilyas M, Oliva R, et al. *Phytophthora infestans* effector AVRblb2 prevents secretion of a plant immune protease at the haustorial interface. Proc Natl Acad Sci. 2011;108: 20832–20837. doi:10.1073/pnas.1112708109

2. Oh S-K, Young C, Lee M, Oliva R, Bozkurt TO, Cano LM, et al. In Planta Expression Screens of *Phytophthora infestans* RXLR Effectors Reveal Diverse Phenotypes, Including Activation of the *Solanum bulbocastanum* Disease Resistance Protein Rpi-blb2. Plant Cell. 2009;21: 2928–2947. doi:10.1105/tpc.109.068247
