# Supplementary material for: Subfunctionalization of NRC3 altered the genetic structure of the Nicotiana NRC network
Source: PLoS Genet. 2024 Sep 12;20(9):e1011402. doi: 10.1371/journal.pgen.1011402 (PMC11421798; doi:10.1371/journal.pgen.1011402)
Supplement: S4 Table — (PDF) [file pgen.1011402.s026.pdf]

S4 Table. List of constructs used in cell biology assays

Vector backbone	Promoter	protein name	Tag	OD_{600}	Reference
pGWB12	35S	AVRblb2	N terminal flag	0.1	[1,2]
pT50	35S	AvrPto	C terminal flag	0.2	[3,4]
pGWB555	35S	RFP	none	0.2	[1]
pICH47751	35S	Rpi-blb2	none	0.2	This study
pK7WG2	35S	NbSOBIR1	C terminal mCherry	0.2	[5]
pICH86988	35S	$NbNRC4^{AAA}$	C terminal GFP	0.2	This study
pTFS40	35S	Pto	C terminal GFP	0.5	[3,4]
pICH86988	35S	$NbNRC3^{L21E}$	C terminal GFP	0.5	This study
pICH86988	35S	$SlNRC3^{L17E}$	C terminal GFP	0.5	This study
pICH86988	35S	$N^{L21E}SS$	C terminal GFP	0.5	This study
pICH86988	35S	$N^{L21E}N_3N_{T5be}$	C terminal GFP	0.5	This study
pICH86988	35S	$N^{L21E}N_{PKK}N_{THK}$	C terminal GFP	0.5	This study

References

1. Bozkurt TO, Schornack S, Win J, Shindo T, Ilyas M, Oliva R, et al. *Phytophthora infestans* effector AVRblb2 prevents secretion of a plant immune protease at the haustorial interface. Proc Natl Acad Sci. 2011;108: 20832–20837. doi:10.1073/pnas.1112708109

2. Oh S-K, Young C, Lee M, Oliva R, Bozkurt TO, Cano LM, et al. In Planta Expression Screens of *Phytophthora infestans* RXLR Effectors Reveal Diverse Phenotypes, Including Activation of the *Solanum bulbocastanum* Disease Resistance Protein Rpi-blb2. Plant Cell. 2009;21: 2928–2947. doi:10.1105/tpc.109.068247

3. de Vries JS, Andriotis VME, Wu A-J, Rathjen JP. Tomato Pto encodes a functional N-myristoylation motif that is required for signal transduction in Nicotiana benthamiana. Plant J. 2006;45: 31–45. doi:https://doi.org/10.1111/j.1365-313X.2005.02590.x

4. Rathjen JP. Constitutively active Pto induces a Prf-dependent hypersensitive response in the absence of avrPto. EMBO J. 1999;18: 3232–3240. doi:10.1093/emboj/18.12.3232

5. Li Y-H, Ke T-Y, Shih W-C, Liou R-F, Wang C-W. NbSOBIR1 Partitions Into Plasma Membrane Microdomains and Binds ER-Localized NbRLP1. Front Plant Sci. 2021;12: 721548. doi:10.3389/fpls.2021.721548
