# Supplementary material for: Subfunctionalization of NRC3 altered the genetic structure of the Nicotiana NRC network
Source: PLoS Genet. 2024 Sep 12;20(9):e1011402. doi: 10.1371/journal.pgen.1011402 (PMC11421798; doi:10.1371/journal.pgen.1011402)
Supplement: Attachment — Submitted filename: Response to reviewer Huang et al.pdf [file pgen.1011402.s034.pdf]

Dear Dr. Tiancong Qi,

Thank you for considering our manuscript as a research article in PLOS Genetics.

We have thoroughly reviewed the comments from all three reviewers and revised the manuscript accordingly. Following the suggestion of Reviewer 3, we have changed the title to "Subfunctionalization of NRC3 altered the genetic structure of the Nicotiana NRC network" to more accurately reflect our findings. All three reviewers also provided feedback on the Turbo-ID experiments and the model on transient interactions. Despite our considerable efforts to address their suggestions, the best evidence we could obtain was similar to that in the earlier submission. Consequently, we decided to remove the section on Turbo-ID experiments and the statement on transient interactions. This adjustment allows the manuscript to focus more on the subfunctionalization of NRC3 alleles that altered the sensor-helper NLR compatibility. We believe this presents a compelling story on the genetics and evolution of sensor-helper NLR compatibility and aligns with the scope of PLOS Genetics.

We hope these changes have sufficiently addressed the comments raised by the reviewers. Please see below for point-by-point 'Response to Reviewers'.

We look forward to your response.

Best,
Chih-Hang Wu
Assistant Research Fellow
Institute of Plant and Microbial Biology
Academia Sinica

Reviewer #1: Review of manuscript PGENETICS-D-24-00215

Sensor NLRs, effector-detecting NLRs, in solanaceous plants rely on the presence and function of helper NLRs of the NRC family. This dependency evolved into a highly expanded NRC-network in Solanaceae and Convolvulaceae. In this manuscript the authors wanted to understand the molecular mechanism/basis for the evolution of this complex network architecture. They picked out one NRC helper NLR, NRC3, and studied the functional divergence of various NRC3 variants. The authors phylogenetic analysis provide evidence that NRC3s form three allelic groups with different specificities/compatibilities for sensor NLRs, mainly Rpl-blb2. By the employment of ancestral sequence reconstruction and analysis of natural and chimeric variants the authors identified six (!) amino acids being key in the sensor-helper compatibility in the helper NRC3. These residues lie in the NB-ARC and LRR domain. The authors hypothesize that these residues are important for the difference in a supposed transient interaction (pre- and post-effector sensing) of the sensor with the helper, having a 'stronger or longer' transient interaction between compatible sensor-helper NLR variants. This is based on a TurboID proximity labeling experiment. The authors further show that only compatible sensor-helper combinations lead to the formation of helper NLR puncta at the membrane and thus resistosome formation.
In their final model the authors suggest that the helper NLR, NRC3, homodimer is 'targeted' by the effector-modified/bound compatible sensor and transiently interacts with the identified residues, which are located at multiple surfaces of the helper NLR. These transient interactions lead to the disruption of the helper homodimer and the formation of the helper

sextuple resistosome. The paper is very well written, and the experiment are presented in a very appealing manner and are of great quality!

We thank the reviewer for the positive comments about the writing and quality of the experiments.

The conclusions drawn by the authors make for sure sense, however, I am not so convinced by the difference in the transient interaction of the compatible vs. the non-compatible helper with the Rlp-blb2 sensor.

We thank the reviewer for this comment. A similar comment was raised by the other two reviewers. We have considered the suggestions from all three reviewers and performed new experiments using different experimental designs and more time points. However, the results we obtained were similar to what we previously observed. Given this outcome, we have removed the data and statements related to transient interactions but have included a few points on the potential mechanisms in the discussion. This has then allowed us to focus on the evolutionary aspects of NRC3 subfunctionalisation, an area often overlooked in the area of plant-microbe interactions. We believe even without the transient interaction data, this manuscript still presents a compelling story on the genetics and evolution of sensor-helper NLR compatibility, providing a novel aspect to the current knowledge in NLR evolution.

In Figure 5 D the one lane is their evidence that the transient interaction of compatible NRC3 is stronger/longer upon AVRblb2 expression than the non-compatible, because the increase in biotin labeling is not seen for the incompatible NRC3. I think this is weak evidence. Another closer time point would maybe help to show this enhanced biotinylation visible in more than one lane/sample.

(See the response above)

Another explanation would be that the (transient) interaction strength is not the cause of the incompatibility, but that the effector-bound/modified sensor-NLR is unable to break the homodimer in the incompatible situation, right. I think this possibility is not excluding the 'interaction-strength' hypothesis, and the mechanism could rather be a combination of both. The sensor NLR, upon binding/detection of the effector changed confirmation so that now the interaction with the helper, which also happened pre-effector recognition, can break the homodimer via interaction with the specific herein identified residues, which than leads to NRC activation. The six residues that the authors identified in the NRC3 protein are not required for sensor-helper steady-state interaction, but for the activation process. Thus, the transient interaction – at least in my opinion, is the same for both compatible and incompatible helper NRC3 – for sure pre-effector recognition.

(See the response above)

Minor issues:
Title: functional divergence – is the paper really providing evidence for functional divergence in the meaning of new function or diverged functions of the NRCs? Or is it rather a functional specification in regard to what sensor NLRs can use which NRC? I am bringing this up as a non-evolutionary biologist, because for me functional divergence was rather associated with a neo-functionalization. Well, as I said, this way of thinking of it may be wrong and I would be happy to be corrected and to be able to learn something new.

Functional divergence includes neofunctionalization, subfunctionalization, and, in a broader definition, nonfunctionalization. In the research community of plant-microbe interactions, neofunctionalization is more often mentioned since many groups are looking for a novel recognition spectrum or novel functions of effectors, whereas the contribution of subfunctionalization and nonfunctionalization in the plant-microbe interactions are often overlooked. Therefore, we believe our manuscript offers a unique and compelling perspective by highlighting the roles of subfunctionalization and nonfunctionalization in the evolution of plant-microbe interactions. As suggested by reviewer 3, we have revised the title to “Subfunctionalization of NRC3 altered the genetic structure of the *Nicotiana* NRC network”.

Line 52: Is it really shown for plant NLRs that they hydrolyze ATP? Or is it rather so that they can bind both ADP and ATP/dATP?

We thank the reviewer for pointing this out. While it was proposed to hydrolyze ATP a long time ago, recent data suggest that the bound nucleotide identity is more relevant. We have rephrased the sentence.

Lines 197: To which NRC3 clade (a, b or c) does PinNRC3 belong to?

Based on the phylogenetic tree, the PinNRC3 does not belong to any of the three clades defined here. We have now also replaced the phylogenetic three in Fig. 1C to clarify this.

Figure 5:
Could the authors do the same experiment in presence of protease inihibitors? Maybe this would also help to see a potential difference in biotinylation even better. Maybe the time points chosen could also be put closer together, lets say 5, 6, 8 and 10 hours?
Why is the biotin labeled Rpi-blb2 not accumulating over time? Is this due to rapid degradation of Rpi-blb2? The potential rapid degradation of Rpi-blb2 makes it difficult to conclude much.
Is the biotin provided enough to also show biotinylation of Rpi-blb2 at the later time points. A least for the incompatible NRC3 it should be the same over time, no? Is it possible to co-express NRC3 with another tag to show biotinylation of this NRC3 (in the homodimer and/or the resistosome) in the samples?

(Please see the response above, this part has now been removed)

Material and methods:
Cell death quantification – It would be nice to indicate what region of the infiltrated region was used for quantification. What is exactly meant by “Cell death intensity was normalized by dividing the pixel value by the maximum pixel value of the signal (65535).” Is (65535) regarding to a reference?

We circle the whole infiltrated area for the quantification. The number 65535 is the maximum signal intensity at each pixel that the machine can detect. If the signal value is close to this number, it means the exposure is saturated, which should be avoided by reducing the exposure time.
We rephrased the sentence to “Cell death intensity was normalized by dividing each pixel value by the maximum signal pixel value (65535) detected by the UVP ChemStudio”. We hope this is now more clear.

Reviewer #2: (The review is not uploaded as an attachment.)

In the manuscript, Huang and Huang et al. provide a compelling analysis of both natural and chimeric variants of NRC3, highlighting crucial amino acids involved in sensor-helper compatibility and offering insights into the evolutionary dynamics of the NLR network. Despite the strengths of the study, I believe its impact could be further enhanced by addressing certain areas through additional experiments and discussions.

Major Concerns:

1. The use of AlphaFold2 for predicting the structure of the NRC3 homodimer is acknowledged. However, the absence of experimental validation to confirm NRC3's dimerization in its resting state is a significant oversight. Given the complex nature of protein interactions and the crucial role of oligomer states in protein functionality, integrating experimental evidence for the predicted dimerization would substantiate the findings. Employing Blue Native PAGE analysis, similar to the approach for NRC2 homodimer studies, could offer the needed validation, thereby solidifying the structural assumptions presented.

We thank the reviewer for this suggestion. We have now included BN-PAGE data of NRC3 in Fig. 6C. Indeed, NRC3 migrates as homodimers in the resting state, and forms oligomers at the activated state, similar to recently reported cases of NRC2 and NRC4.

2. The manuscript intriguingly marks key natural mutations on the predicted model in Figure 4, leading to a section rich in predictions and hypotheses. I recommend relocating this part to the discussion section of the manuscript. This move would not only clarify the distinction between the results and speculative interpretations but also provide a more appropriate context for exploring the implications of these findings.

We thank the reviewer for this suggestion. We decided to keep the AlphaFold predicted results in the main figure (now Fig 5) and include a few more cell death assays to test the hypothesis that multiple surfaces are involved in sensor-helper compatibility. We have removed the section rich in hypothesis from the result section. This hypothesis mentioned originally about the interaction is still discussed in the discussion section. We hope the reviewer agrees with these changes.

3. The innovative application of TurboID for probing transient protein interactions is commendable. Yet, the interpretation of the labeling results warrants further scrutiny. The reported pattern of a strong biotin-labeled signal at 6 hours post-treatment, diminishing at 12 hours and disappearing by 30 hours, calls for a deeper exploration into the stability and fate of the proteins initially detected. A discussion on potential rapid degradation or other processes affecting the observed signal dynamics would be valuable. Additionally, referencing the initial TurboID application in plants (PMID:31535972), which showed progressively stronger signals from 15 minutes to 3 hours post-treatment, could provide a benchmark for these results. Such a comparison might illuminate whether the differences observed are due to experimental design or specific protein behaviors, offering a richer interpretation of the data.

We thank the reviewer for this comment. A similar comment was raised by the other two reviewers. We have decided to remove the TurboID experiment and statement on the transient interaction (see the response above to reviewer 1). In this way, we can focus more on the rest of the story and only one paragraph about the mechanisms remains in the discussion. We hope the reviewer agrees with this change.

Reviewer #3: Ching-Yi Huang and colleagues present a body of work investigating the determinants responsible for functional divergence conditioning signaling specificity in a helper-sensor NLR network constituting the plant immune system in Solanaceae plants. This work conceptually makes an incremental contribution to our understanding on how NLR network has evolved to modulate signaling, initiated from hypervariable NLR sensors, funneling through a group of helper NLRs. In the past, the expansion of NRCs in the clade of Solanaceae had been studied in detail to match the signaling partners of handful sensor NLRs, covering a wide range of pathogens as entry points. This work focused on the compatibility of one of the NRC groups, NRC3 clade, and its cognate pair sensor NLR, Rpi-blb2, since the signaling partnership of this sensor-helper pair is on and off across the Solanaceae species, for example in tomato and tobacco lineages. The level of execution in their investigation of the compatibility of sensor-helper NLR is quite exquisite; the authors were able to 1) narrow down the causal residues contributing to the specificity down to six via an extensive series of domain-swapping followed by mutation analyses and quantitation of HR assays in N. benthamiana, 2) succeeded to experimentally address ancestral function in regards to the compatibility using the synthetic, ancestral NRCs, 3) provide molecular explanation on the time course scenarios of helper-dimer activation towards high-order oligomers, inferred from Turbo-ID experiments and cell biological assessment of the punctate formation on the membrane. The series of data provided convinces this reviewer of the mode of action of NRC3 variants, supporting the notion of functional divergence of NRC3 in splitting a subnetwork in terms of fine-tuning sensor choices.

We thank the reviewer for very carefully reading the manuscript and highlighting the positive aspects of the manuscript. We hope this revised version has sufficiently addressed the concerns raised by the reviewer.

While the breadth of data is quite amazing and complete with room for minor improvements, the authors might need to spend some other time to scaffold their findings in the context of whole NRC network, if the authors' main point is to talk about network architecture. In other words, the current presentation and writing shall improve to clarify academic novelty of this work. As alluded in the beginning of this report (with a comment on incremental finding), the authors are encouraged to restructure the introduction to compare this finding to the already published NRC functional divergence. Elaboration on the NRC3 lineage presented in this work is well appreciated; however, the title and overall discussion points on functional divergence of NRCs might not sound new to fill the gap of knowledge. There must be an excitement of the new knowledge built from the currently presented new dataset, which is at the moment difficult to find. If the authors want to highlight the mode of operation of NRC3 variants as a new finding with discovering how functional divergence can be made over evolutionary time-scale, the focus of the manuscript shall be repackaged to convey the strength of this beautiful, extensive work.

We thank the reviewer for this constructive feedback. Please see below for our responses.

With the above-mentioned notion, detailed comments are provided below.

• The title does not fully convey the main findings in the manuscript but remains quite vague and overstretched. The network is not even presented as a main figure either for readers to assess how functional divergence affected the "architecture" of network. Even with the sup figure, I would not call this as an architecture. This is just one layer of a network, being separated by sub-functionalization, which is quite common. To avoid a confusion that it might cause in systems biology, title revision is highly recommended. The current title might not be the strongest point of this work, given that functional divergence of sub-NRC clades were already reported extensively.

We thank the reviewer for raising this point. We have revised the title to "Subfunctionalization of NRC3 altered the genetic structure of the Nicotiana NRC network". We believe this title now more precisely reflects the main finding of our manuscript. While we have previously reported on several sub-NRC clades exhibiting different helper NLR activities or novel functions, none of our past work has focused on the molecular evolutionary aspect of NRC functional divergence. To our knowledge, this is the first time the subfunctionalization event of NLR proteins has been described and mapped at the residue level (if I did not miss any literature). While events of neofunctionalization, such as acquiring new recognition spectra or novel virulence activities, are frequently discussed within the molecular plant-microbe interactions research community, the role of subfunctionalization in the long-term evolution of the plant immune system and plant-microbe interactions has been largely overlooked. We believe this is the unique and novel aspect of our current manuscript.

• Issues in regards to Turbo-ID experiment
Induction of the sensor NLR expression assessed by western blot peaked at 12hours post induction as shown in Fig 5B. However, the IPed pool of Rpi-blb2 at 6 hours vs. 12 hours in the panel D is quite different with much more intensity found in the 6-hour samples. I wonder whether the faint detection of Strep-decorated sensor NLRs in 12-hour panel would be due to the less efficient IP condition. I wonder the claim of this "transient" interaction based on this data quality could be justified. Could it be just associated with rapid turn-over of the sensor NLR?
Another notion is that biotin-treatment was carried out at one point, namely at the induction point, and once a protein is biotinylated, it remains as biotinylated. To really address "transient" nature of the proximity labeling, the same duration shall be given between the biotin-labeling and sampling time.
In addition, L370-377: description shall be better made to explain whether or not the Streptavidin-decorated sensor NLR is from the IPed sample.

We thank the reviewer for this comment. A similar comment was raised by the other two reviewers. We have decided to remove the TurboID experiment and statement on the transient interaction (see the response above to reviewer 1). In this way, we can focus more on the rest of the story and only one paragraph about the mechanisms remains in the discussion. We hope the reviewer agrees with this change.

• A scaffolding of knowledge and pre-existing information shall be better addressed. Here I pinpoint several places where changes would be beneficial.
1) Introduction of the functional diversification of NRCs across different lineages shall be better explained starting from Fig. S1. NRCs themselves have undergone duplication events

followed by functional diversification. How much is known about the ancestral function basal to NRC1/2/3/X vs. NRC 4-9 lineage? It would be much nicer to scaffold the information of NRC evolution in a broad context.

We thank the reviewer for this comment. At the moment, we know the NRC network likely originated from a helper-sensor gene cluster similar to NRC0-H/NRC0-S reported in Sakai et al and Goh et al (PMID: 38833594, 38922300). We have elaborated on this in the introduction to scaffold the NRC evolution in a broad context. However, we do not know much about the ancestral function basal to NRC1/2/3/X vs. NRC 4-9 lineages. The evolutionary history together with functional data of NRC evolution from a common ancestor to ancestral NRC1/2/3/X and ancestral NRC4-9 has not been fully reconstructed. The NLRome datasets (https:/doi.org/10.5281/zenodo.10354350) and ancestral reconstruction datasets (https:/doi.org/10.5281/zenodo.1036019) related to the current manuscript may serve as a starting point for addressing this question.

2) It would be really helpful if the authors could further articulate the reasons of picking the NRC3 clade to further elaborate network topology, which seems to be coming from the result in Fig. S2. What is the relative position of NRC3-driven network in the whole NRC immune network? Is it a part of the network particularly prone to show peripheral modification, if the authors talk about architecture?

We thank the reviewer for this comment. We believe this sentence “We reasoned that exploring closely related NRC homologs that show different compatibilities to the sensor NLRs may shed light on the molecular determination and evolution of the sensor-helper compatibility.” justifies the rationale for our target selection. The NRC3 group was the only group we found clear differences between alleles, prompting us to initiate this study.

Moreover, in a recent study, NRC3 alleles were shown to be more divergent in the interfaces targeted by the pathogen effector SS15, whereas NRC1/2 alleles were more conserved (Sugihara et al., 2024 https://doi.org/10.1101/2024.06.16.598756). This suggests that NRC3 is probably more prone to modification during evolution. However, NRC4 alleles show even greater diversity when compared to the other three NRC (1/2/3) groups, yet dissecting this is beyond the scope of the current study.

3) When the NRC helper network is first introduced in L58-60, it would be advisable to indicate where as it operates, such as within Solanaceae, instead of indicating it later (e.g. L66).

We thank the reviewer for this comment. We now added “asterids” in the sentence as two of our new stories extended the studies of NRC-H and NRC-S to the species beyond Solanaceous plants (Sakai et al., 2024 and Goh et a., 2024). The “Solanaceae” in Line 66 remains there as we described specific NRC homologs from Solanaceae in that paragraph.

4) Figure 3 itself and the legend alone cannot indicate which color of the bar indicate which protein. Fig. S8A should be moved to the main to inform the readers.

We thank the reviewer for this suggestion. We have now indicated the color in the figure legends and moved Fig. S8A to Fig. 3A.

5) The location of six residues (Fig. S17A) shall be introduced in the main figure. From L308 on when the position in number begins to be introduced, till the accompanying text of Fig. 4, the explanation relies on the Sup S17A .

We thank the reviewer for this suggestion. We have now combined part of Fig S16, Fig S17, and Fig S18 into the new Fig. 4. We hope this is clearer.

6) L71-73 semi-colon usage seems more adequate grammatically, as current clauses lack a conjunction in some places and the meaning is not clear. For example, was the work of Prf and Rx tested also in N. benthamiana?

All of those have been tested in *N. benthamiana*. So, we now moved "in the model solanaceous plant species *Nicotiana benthamiana"* to the end of the sentence.

7) Fig S7 and accompanying test in the main: please clarify what the curation means. It would be good to see an alignment with SlNRC3 to compare this distant copy to the focal one.

We thank the reviewer for this suggestion. We have now added SlNRC3 into the alignment in Fig. S7 and rephrased the sentence.

8) Fig. S5: ancestral reconstruction of these NRC sequences would heavily depend on the quality of the phylogenetic trees. At least, aligning them with the focal ones, Sl and Nb NRCs would point out a distinct or highlight the places with high divergence.

We thank the reviewer for this suggestion. We have now included SlNRC3a, NtNRC3b1 and NbNRC3c in the alignment in Fig S5.

Minor textual comments
L141: "functional divergence has evolved" shall be revised. It is the NRC3 homologs that have evolved to become functionally divergent.

We thank the reviewer for this suggestion. We have rephrased the sentence.

L176: rephrasing is recommended, such that "which molecular events contributed to functional divergence of NRC3".

We have now rephrased the sentence.

L192: this group of NRC3 has "been" nonfunctionalized…

We have now modified it accordingly.

L189-193: this result should be explained with indication of N88 DV used as a positive control.

We have now added N88DV in the text and clarified this.

L197-203: without seeing Fig. S24, it was impossible to obtain information about Petunia NRC3. Please indicate where it belongs.

Petunia NRC3 is sister to the theer allelic groups mentioned here. We have now added "which is sister to the three NRC3 allelic groups mentioned above (Fig. 1*C*)" to the text.

L284-5: Not all NRCs in Fig.1C is "tested above" experimentally.

We have now changed to "mentioned" above.

L370-372: isn't it that the Rpi-blb2 decoration with Streptavidin was detected after IPing? The text shall align with the experimental procedure.
The epitope tag flag should be capitalized throughout the manuscript.

This has now been removed. Please see the response above. No epitope tag Flag is mentioned in the manuscript now.
